# Asymmetrical Shoulder: A Possible Sign of Supraclavicular Lymphadenitis in Children

**DOI:** 10.7759/cureus.89831

**Published:** 2025-08-11

**Authors:** Megumi Akutsu, Daisuke Matsubara, Tadahiro Mitani, Tomoyuki Ota, Yuji Gunji

**Affiliations:** 1 Department of Pediatrics, International University of Health and Welfare, Nasushiobara, JPN; 2 Department of Pediatrics, Jichi Medical University, Shimotsuke, JPN; 3 Division of Community and Family Medicine, Jichi Medical University, Shimotsuke, JPN; 4 Department of Radiology, International University of Health and Welfare, Nasushiobara, JPN

**Keywords:** asymmetrical shoulder, cellulitis, magnetic resonance imaging, skip lesions, supraclavicular lymphadenitis

## Abstract

We report a rare case of pediatric supraclavicular pyogenic lymphadenitis presenting with an "asymmetrical shoulder" in a five-year-old boy. The boy developed right neck and shoulder pain following the resolution of cellulitis at the site of insect bites on his right forearm. Despite the absence of visible inflammation in the shoulder or proximal lymph nodes, short tau inversion recovery magnetic resonance imaging (STIR-MRI) revealed an enlarged supraclavicular lymph node with surrounding edema. He fully recovered following intravenous antibiotic treatment. This case highlights that lymphadenitis in remote drainage sites, such as the supraclavicular nodes, may develop even after the resolution of distal cellulitis at the primary site. Notably, in the present case, inflammation was absent in the anatomically proximal lymph nodes (such as those at the elbow or subclavicular lymph nodes) and appeared only in the supraclavicular region. Lastly, the asymmetrical shoulder appearance may serve as a valuable clinical sign of the underlying lymphadenitis. We also discuss key considerations when encountering supraclavicular lymphadenitis, which carries a high risk of malignancy at all ages.

## Introduction

Acute cervical pyogenic lymphadenitis commonly occurs in children, most often caused by upper respiratory tract infections [1]. Although less frequent, lymphadenitis may also result from skin/soft tissue infections such as cellulitis [2, 3]. Among these, supraclavicular lymphadenitis, which is often associated with malignancy, is a rare manifestation in children [3]. Here, we present a unique pediatric case of supraclavicular pyogenic lymphadenitis characterized by several distinct clinical features: the appearance of an "asymmetrical shoulder," with both topological and chronological "skips," following cellulitis of the extremity due to an insect bite. We also discuss important considerations when encountering supraclavicular lymphadenitis.

## Case presentation

Six days prior to presentation, a previously healthy, obese five-year-old boy (body mass index: 24 kg/m2, 99.8th percentile of the reference population per the CDC) sustained insect bites on his right forearm while playing in the park during early summer. He developed local inflammation around the area, although he did not scratch the site. The boy gradually began experiencing additional pain around his ipsilateral neck/shoulder, with no visible skin changes in the affected area. On the following day, a family physician diagnosed him with local cellulitis around the site of the insect bite. Two days later, oral amoxicillin and antipyretics temporarily relieved his fever and the local inflammation on his right forearm. However, his fever relapsed, and his neck/shoulder pain worsened, following which he was admitted to our institute.

**Figure 1:**
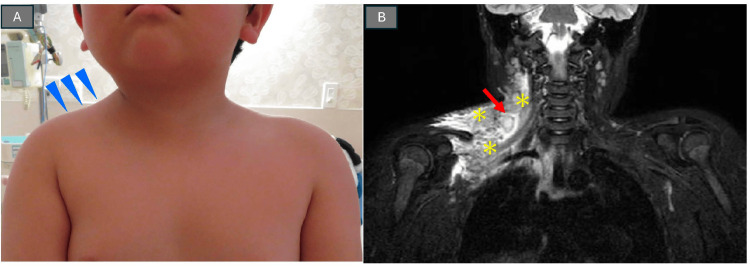
Images of an asymmetrical shoulder in a five-year-old boy suggestive of supraclavicular lymphadenitis caused by an insect bite on the ipsilateral distal extremity (A) Anterior chest photograph showing right shoulder swelling and the resulting “asymmetrical shoulder” appearance. (B) Coronal STIR-MRI imaging corresponding to (A), demonstrating an enlarged, hyperintense right supraclavicular lymph node (red arrow), with surrounding subcutaneous edema (asterisks), leading to the shoulder swelling.

On admission, his vital signs included: body temperature, 39.7 °C; blood pressure, 92/48 mmHg; pulse rate, 138 bpm; respiratory rate, 24 breaths per minute; and oxygen saturation, 97% on ambient air. He appeared anguished from the pain and was unable to move his right shoulder, which was noticeably swollen, giving an "asymmetrical shoulder" appearance (Figure 1A). No skin changes were observed around his shoulders or limbs, and his right forearm showed resolution of earlier cellulitis. No lymph node enlargement was observed in the neck, armpits, elbows, or groin. No hepatosplenomegaly was observed. Moreover, he had no respiratory symptoms, throat redness, or untreated caries. He had no recent cat exposure or a history of international travel. Although the insect involved could not be identified, insects in this area are not typically known to cause severe symptoms such as those observed in rickettsial infections.

Laboratory data showed marked inflammation, with a white blood cell count of 29,300/µL (91% neutrophils) and C-reactive protein of 7.7 mg/dL, with a normal platelet count (Table 1). A throat swab culture grew α-hemolytic streptococci, while the blood culture was negative. Cervical lymphadenitis secondary to throat infection, possibly caused by group A *Streptococcus*, was initially suspected. Short TI (tau) inversion recovery magnetic resonance imaging (STIR-MRI) was performed to assess tissue edema and lymphatic stasis, which revealed an enlarged and hyperintense right supraclavicular lymph node (Figure 1B, red arrow) with surrounding subcutaneous edema (Figure 1B, asterisks), causing shoulder swelling. There was no abscess formation or signs of lymphangitis in the right arm. The patient was diagnosed as having right supraclavicular pyogenic lymphadenitis.

**Table 1:** Laboratory values on admission

Parameters	Values	Reference Range
White blood cell (×10^3^/µL)	29.3	3300-8600
Neutrophil (%)	90.5	42-74
Hemoglobin (g/dL)	12.9	13.7-16.8
Platelet counts (×10^4^/µL)	35.5	15.8-34.8
C-reactive protein (mg/dL)	7.74	0-0.14
Albumin (g/dL)	3.5	4.1-5.1
Total-bilirubin (g/dL)	0.5	0.4-1.5
Aspartate aminotransferase (U/L)	23	13-30
Alanine aminotransferase (U/L)	16	10-42
Lactate dehydrogenase (U/L)	214	124-222
Creatine phosphokinase (U/L)	38	59-248
Blood urea nitrogen (mg/dL)	8	8－20
Creatinie (mg/dL)	0.37	0.65-1.07
Sodium (mmol/L)	134	138-145
Potassium (mmol/L)	4	3.6-4.8
Chlorine (mmol/L)	99	101-108

Although no specific pathogen was identified, empiric treatment with ampicillin sodium/sulbactam sodium (150 mg/kg/day) for 10 days resulted in clinical resolution. Given the favorable response to antibiotic therapy, we decided not to perform a lymph node biopsy. A follow-up MRI on the tenth day of hospitalization showed normalization of the previously enlarged right supraclavicular lymph node. Four weeks later, a negative anti-streptolysin O test ruled out active group A *Streptococcus* infection. Pyogenic supraclavicular lymphadenitis was most likely secondary to cellulitis, possibly from the insect bite on the forearm. The patient remained healthy without any recurrence one year after discharge. 

## Discussion

We report a rare condition of supraclavicular pyogenic lymphadenitis in a child, presenting with an "asymmetrical shoulder" appearance, possibly secondary to cellulitis on the ipsilateral extremity. This case highlights several important clinical insights. First, an asymmetrical shoulder may reflect unilateral swelling, partially caused by supraclavicular lymphadenitis. Because the supraclavicular lymph node is often difficult to palpate, particularly in obese patients, this "asymmetry" may be the only manifestation and can easily be overlooked without thorough examination. This highlights the role of STIR-MRI in assessing lymphadenitis and related soft tissue changes [4]. Second, by the time the asymmetrical shoulder became apparent, the insect bite on the extremity and its associated cellulitis had almost resolved, which could have led to a missed diagnosis. Prior administration of oral antibiotics may have masked the clinical manifestation. This case emphasizes the fundamental clinical concept that infection or inflammation can cause lymphadenitis in the corresponding (draining) lymph nodes, even after the primary lesion has healed. Third, only the supraclavicular nodes, the "final" lymphatic drainage points, manifested lymphadenitis, bypassing the involvement of more anatomically proximal nodes (such as those at the elbow or subclavicular lymph nodes) that typically drain the affected extremities. Finally, supraclavicular lymphadenitis may also be associated with malignancies or systemic diseases (e.g., lymphoma and lupus), given that these nodes drain from broad regions, including the neck, upper limbs, and chest [5]. At all ages, supraclavicular lymphadenitis is associated with a high risk of malignancy, including leukemia, lymphoma, and histiocytosis, with reported rates as high as 75% [6]. Right-sided supraclavicular lymphadenitis is often associated with malignant involvement of mediastinal lymph nodes. In contrast, left-sided supraclavicular lymphadenitis, commonly referred to as Virchow’s lymph node, may indicate not only mediastinal disease but also intra-abdominal malignancies, most frequently lymphoma or metastatic disease such as neuroblastoma [3]. Although the final diagnosis in the present case was infectious supraclavicular lymphadenitis, further invasive procedures might have been required to rule out alternative causes if the clinical response had not been favorable. 

A systematic review of seven studies (2687 children with enlarged lymphoid tissue) to identify the etiology of cervical lymphadenopathy found that no specific diagnosis was made in approximately 70% of cases [7]. In this context, in the absence of an identified causative pathogen in the present case, a direct connection between supraclavicular lymphadenitis and cellulitis of the extremity could not be definitively established. However, the typical progression of symptoms - specifically, the acute onset of unilateral neck/shoulder pain shortly after insect bites on the ipsilateral extremity - suggests a possible link, likely owing to microorganisms entering the lymphatic system at the bite site [8]. In addition, the favorable response to antibiotics supports an infectious cause, likely involving skin bacteria, such as methicillin-susceptible *Staphylococcus aureus* or *Streptococcus pyogenes*, both of which can reasonably account for the acute presentation of lymphadenitis developing over several hours to days [1, 3]. Cat-scratch disease or mycobacterial infection can be ruled out due to the absence of a relevant clinical history or the progression of clinical course, which typically presents with subacute or chronic symptoms [1, 3]. 

We previously observed inguinal lymphadenitis secondary to a knee injury, where thigh lymphangitis appeared later, disrupting the typical drainage flow (knee → thigh → inguinal), a concept as "topological skip" [9]. The present case may illustrate both "topological" and "chronological" skips, wherein supraclavicular lymphadenitis appeared to bypass the anatomically corresponding lymph nodes (such as those at the elbow or subclavicular lesions), and became apparent only after the resolution of cellulitis on the extremities.

## Conclusions

We present a rare pediatric case of supraclavicular pyogenic lymphadenitis, presenting with an "asymmetrical shoulder" which was clearly visualized on STIR-MRI. This "asymmetrical shoulder' sign, reflecting unilateral swelling, can serve as a clinical indicator of supraclavicular lymphadenitis, even in the absence of overlying skin changes. Furthermore, clinicians should be aware that supraclavicular lymphadenitis - often associated with malignancy - may stem from a distal infection that has already resolved and may bypass the anatomically proximal lymph nodes.
